# Harnessing electronic data to inform health decisions

**DOI:** 10.1093/jamiaopen/ooz021

**Published:** 2019-06-28

**Authors:** Indra Neil Sarkar

**Affiliations:** Center for Biomedical Informatics, Brown University, Box G-R, Providence, Rhode Island 02912, USA

Electronic health data have become a mainstay in the care and biomedical research. The electronic health record (EHR) serves as an aggregator of data from myriad health data sources, forming the basis for clinical documentation and supporting communications between members of the health team (including the patient!). In research contexts, the EHR is often complemented with electronic data capture systems. Collectively, the clinical and research enterprise is making significant strides in reducing the reliance on paper-based systems, thus supporting the longer-term goals of reproducibility and reuse.

The ability to generate volumes of data and unprecedented rates comes with a new set of challenges, as described in many of the articles in this issue. More data does not necessitate higher quality, and so it is essential that we continue to invest in development of ways to leverage data as they are gathered and support their transformation into usable information. Furthermore, it is paramount that the clinical and research community consider the implications of how electronic health data systems, whether focused on clinical or research, can affect interactions with patients and others in the clinical or research ecosystem.

Clinical practice and research insight depend on a balance of human intelligence and machine guided insights. This is clearly exemplified by the pioneering work that continues to be done by the nursing informatics community. I was pleased that *JAMIA Open* was able to join *JAMIA* in celebrating Nurses Week (May 6–12, 2019) by identifying publications showcasing nursing informatics work: https://academic.oup.com/jamiaopen/pages/nurses_week_collection.

Joint collections that span *JAMIA* and *JAMIA Open* reflect the strong relationship between the sibling journals. The ability to contribute to such special collections also reflects the continued maturation of the journal and defining its role in the biomedical and health informatics publication landscape. I remain grateful to the contributors, reviewers, and editorial board members for their commitment that has yielded a journal that is built around high-quality work and advancements in our discipline.

